# Inflammatory cytokines are associated with response and prognosis in patients with esophageal cancer

**DOI:** 10.18632/oncotarget.17671

**Published:** 2017-05-07

**Authors:** Susanne Blank, Henrik Nienhüser, Lena Dreikhausen, Leila Sisic, Ulrike Heger, Katja Ott, Thomas Schmidt

**Affiliations:** ^1^ Department of General, Visceral and Transplantation Surgery, University of Heidelberg, 69120 Heidelberg, Germany; ^2^ Romed Klinikum Rosenheim, 83022 Rosenheim, Germany

**Keywords:** cytokines, inflammation, prognosis, response, esophageal cancer

## Abstract

**Background:**

Esophageal cancer is often marked by aggressive tumor growth and poor prognosis. Patient groups who benefit from perioperative therapy are not yet defined. The tumor microenvironment and circulating factors as possible predictors of response and prognosis gain interest. This study aimed to investigate cytokines in patients’ serum and tumor tissue with regard to response and prognosis.

**Results:**

Median survival between SCC and AC was not different (published previously). Lower levels of CCL11 (Eotaxin-1) and CXCL10 (IP-10) in the tumor tissue were associated with a better prognosis (*p* = 0.022; *p* = 0.002). In the AC subgroup higher concentrations of TGF-β3 in serum and corresponding tumor tissue were associated with adverse prognosis (*p* = 0.035; *p* = 0.006). An association with histopathological response was found for IL-12(p70) and CXCL10 in patients’ sera (*p* = 0.041; *p* = 0.032). The tissue levels of TGF-β1 and TGF-β2 were significantly lower in histopathological responders than in nonresponders (*p* = 0.033; *p* = 0.007). A similar trend was seen for TGF-β3, without statistical significance (*p* = 0.097).

**Materials and Methods:**

Preoperative serum samples and corresponding tumor tissue (*n* = 54), only serum (*n* = 20) or only tissue (*n* = 4) were collected from patients undergoing surgery for cT3/4 esophageal squamous cell cancer (SCC) (*n* = 34) and adenocarcinoma (AC) (*n* = 44). All samples were taken after neoadjuvant treatment. All patients received perioperative chemo(radio)therapy. Cytokine levels of 17 different cytokines were measured by multiplex immunoassay and correlated with clinicopathological factors.

**Conclusions:**

Two chemokines (CCL11 and CXCL10) in posttherapeutic tumor tissue were associated with prognosis in patients with esophageal cancer, lower levels indicating a better prognosis. Lower levels of TGF-β were associated with better response and prognosis in patients with AC.

## INTRODUCTION

Recent changes in the diagnosis and therapy of esophageal cancer have shown to improve patients’ outcome [1]. However, it remains a disease that is mostly detected in advanced stage and thus associated with poor prognosis. The late time of diagnosis – often not until dysphagia occurs – leads to situations, where surgical resection is often difficult if possible at all [2]. As 5-year survival rates often do not exceed 25% [3], amongst other changes, neoadjuvant therapy has become established in order to improve prognosis. These neoadjuvant therapeutical regimens have shown to provide a benefit in survival for patients with advanced stages of the disease [4–7]. In this context it is important to take into account that only responding patients seem to profit from this therapy and thus possible predictors of response and prognosis recently are of greatest interest [8, 9]. However, response prediction has not yet found its’ way into clinical practice. Also as many patients become older it might be important to find criteria to stratify, which patients should receive neoadjuvant therapy [10]. Recently it has been indicated that factors shaping the tumor microenvironment might influence response to therapy and patients’ prognosis [11]. As important factors, different cytokines influence tumor progression by shaping the environment surrounding the tumor or by directly intervening in cell growth and cell survival [12]. Inflammation, as one important aspect of tumor formation and progression, influences the pattern of prevalent cytokines. This inflammation is not only present when the tumor arises. It plays an important role in keeping up tumor proliferation once the tumor has been established and forms its microenvironment [12]. In advanced tumors pro-inflammatory cytokines, growth factors and chemokines fostering tumor progression are mostly predominant, while cytokines inhibiting tumor growth are often lacking [13–15].

Of these pro-inflammatory cytokines interleukin-1 (IL-1), interleukin-6 (IL-6) and tumor necrosis factor α (TNFα) have been previously described in esophageal cancer and associations with several clinicopathological parameters have been shown [16–18]. For IL-1β and IL-6 an association with patients’ prognosis has been reported in several studies [16, 17, 19].

Furthermore chemotactic cytokines are involved in determining the tumor microenvironment. Induced by proinflammatory cytokines, growth factors and pathologic factors, chemokines influence the infiltration of leukocytes into the tumor, cell growth, survival and angiogenesis [20]. In esophageal cancers a complex network of chemokines seems to influence tumor growth in sometimes contradicting ways [21].

Aim of this study was to investigate relevant cytokines (IL-1Ra, IL-6, IL-12, IL-17, CCL 11 (Eotaxin-1), FGF basic, GM-CSF, IFNγ, CXCL10 (IP-10), MCP-1(MCAF), MIP-1α, MIP-1β, Rantes (CCL5), TNFα, TGFβ-1, -2, and -3.) with regard to patients’ clinicopathological parameters, survival and response to neoadjuvant therapy. As response rates range from 20–50% dependent on the chosen regimen, response prediction could help in clinical practice [8]. Different cytokine profiles predicting response and prognosis could thus support the choice for an appropriate regimen.

## RESULTS

78 patients were included in this study. 44 (56.4%) had developed adenocarcinoma (AC) of the gastroesophageal junction (AEG), 34 (43.6%) esophageal squamous cell cancer (SCC). Patients’ pretherapeutic and postoperative clinicopathological characteristics of this cohort were previously published and are displayed in Supplementary Table 1 [22].

### Association of cytokines with clinicopathological factors

IL-1ra, IL-12, IL-17, FGFb, Mip-1α and Rantes (CCL5) serum levels were associated with the tumor type (*p* = 0.027, *p* = 0.008, *p* = 0.002, *p* = 0.040, *p* = 0.011, *p* = 0.011). Patients with SCC had significantly higher serum levels of IL-1ra, IL-12, IL-17, FGFb, Mip-1α and Rantes. Mip-1β, TGF-β2 and TGF-β3 in the serum were associated with the cT category (*p* = 0.021, *p* = 0.013, *p* = 0.031); FGFb, MIP-1α and TNFα with the cN category (*p* = 0.029, *p* = 0.009, *p* = 0.036). Patients with positive postoperative lymph node status had significantly higher serum levels of IL-6, GM-CSF, MCP-2 (MCAF) and MIP-1β (*p* = 0.049, *p* = 0.019, *p* = 0.027, *p* = 0.025). Higher levels of CXCL10 in the serum were found in patients with R0 resection status (*p* = 0.041). In the tumor tissue, TNFα was associated with tumor type and gender (*p* = 0.049 and *p* = 0.002). Patients with G1/G2 tumors had higher tissue levels of Rantes than patients with G3/G4 tumors (*p* = 0.025). The resection status was associated with tissue levels of FGFb (*p* = 0.038). Correlations of cytokines and clinicopathological factors are shown in Table 1.

**Table 1A:** Association of cytokine levels in the tumor tissue and clinicopathological factors

Characteristics		n	IL-1Ra	IL-6	IL-12	IL-17	CCL11	FGF b	GM-CSF	IFN-γ	CXCL-10	MCP-1	MIP-1α	MIP-1β	RANTES	TNF-α	TGF-β1	TGF-β2	TGF-β3
Gender	male	69	2742.0	34.5	10.3	14.7	50.1	430.6	69.9	28.9	168.7	58.5	3.1	48.5	1282.9	15.4	86.3	28.5	9.1
	female	16	8980.3	28.3	14.3	14.7	65.8	283.3	64.6	37.3	632.3	57.1	3.7	46.6	1017.2	20.9	45.9	18.8	4.8
Localization	AEG I/II	44	3178.5	27.1	11.1	16.1	42.6	324.3	69.3	28.9	148.9	57.1	3.1	53.7	1182.6	13.8	86.3	27.8	7.8
	SCC	34	5410.6	45.8	10.9	14.7	51.0	430.6	70.3	28.9	217.3	58.5	3.7	46.6	1262.7	22.5	72.2	28.0	8.7
Grading	G1/2	32	3856.7	28.3	10.9	14.7	42.6	357.8	71.0	28.9	247.0	58.5	2.3	46.6	1962.6	16.2	73.9	28.0	8.0
	G3/4	38	4414.8	27.1	11.1	14.7	58.2	339.8	66.6	28.9	148.9	49.2	3.1	48.5	918.8	17.0	86.0	27.3	10.8
cT Category	cT1	0																	
	cT2	5	8980.3	139.8	9.8	19.7	13.6	344.3	69.9	28.9	43.0	103.5	2.4	26.5	1917.8	19.4	196.3	43.8	7.7
	cT3	69	3856.7	31.3	11.1	14.7	50.1	353.2	70.1	28.9	203.2	58.5	3.3	48.5	1220.5	17.0	73.9	28.0	8.0
	cT4	4	3388.1	15.8	18.1	18.1	84.7	405.9	66.8	26.2	265.5	29.3	7.7	65.8	1617.8	18.5	101.2	28.9	13.3
cN Category	cN0	17	1840.4	41.9	9.8	14.7	37.8	459.1	69.3	28.9	247.0	69.2	2.3	48.5	1017.2	19.4	72.2	28.5	11.1
	cN+	61	3962.3	25.8	11.3	14.7	53.4	344.3	70.1	28.9	158.0	50.3	3.7	63.2	1262.7	17.0	81.8	27.8	5.9
cM Category	cM0	67	3909.5	32.2	10.7	14.7	46.4	348.8	69.3	30.7	210.2	57.8	2.5	46.3	1241.6	17.2	80.6	28.2	8.7
	cM1	11	2966.4	21.0	19.5	15.0	54.6	348.7	70.0	28.0	113.0	56.3	5.1	80.0	1316.2	11.8	80.7	27.1	5.2
pT Category	pT0	17	3962.3	25.8	10.5	13.2	24.1	357.8	69.3	23.5	75.9	38.1	2.1	24.4	952.9	18.6	21.4	18.1	3.8
	pT1	8	3088.2	127.6	12.5	18.0	87.2	403.5	70.2	27.0	158.9	150.5	5.5	106.4	1518.3	23.6	111.5	37.7	3.7
	pT2	10	5552.4	28.1	10.2	16.9	71.0	375.6	72.2	27.1	230.1	55.7	3.1	44.7	1640.2	15.7	75.5	27.6	13.1
	pT3	38	4135.7	31.1	11.2	15.4	55.8	324.6	65.8	32.4	348.6	61.5	3.8	83.5	1388.2	16.2	87.2	31.1	12.0
	pT4	4	897.2	15.1	8.2	12.5	11.6	273.6	74.1	28.9	25.2	14.9	2.2	19.8	169.7	10.6	86.0	48.2	23.3
pN Category	pN0	36	3585.6	50.1	9.8	14.7	42.6	406.9	70.3	28.9	158.0	72.8	2.3	48.5	1282.9	19.4	81.8	30.4	8.0
	pN+	40	3856.7	24.8	11.3	16.1	53.4	310.7	66.6	32.4	331.5	49.2	3.7	77.9	1182.6	15.4	79.3	27.0	9.4
pM Category	pM0	74	3909.5	30.2	11.0	14.7	50.6	348.7	69.6	28.9	199.3	57.8	3.2	48.5	1241.6	17.2	76.6	28.0	8.0
	pM1	3	2543.5	29.5	14.7	25.6	32.9	535.9	55.0	30.7	266.1	68.0	12.0	60.6	2105.4	11.8	134.8	31.4	16.9
Resection status	R0	68	3585.6	29.1	11.3	14.7	53.4	406.9	70.1	28.9	203.2	58.5	3.1	48.5	1262.7	17.0	81.8	28.0	7.8
	R1/2	10	5595.3	33.0	9.8	14.7	31.9	221.4	65.0	32.4	195.4	38.5	3.9	89.0	584.6	18.6	72.2	30.4	10.8
Response																			
Clinical	Resp.	21	3962.3	28.3	11.3	14.7	39.1	344.3	70.3	25.4	75.9	38.5	2.4	26.5	1118.7	20.9	81.8	27.8	7.7
	Nonresp.	55	3523.7	30.2	10.8	16.3	52.2	365.9	68.9	32.4	244.9	57.8	3.7	51.1	1338.3	15.8	82.8	28.2	10.8
Histopathological	Resp.	26	3190.7	29.1	11.1	16.1	51.0	325.0	69.3	28.9	247.0	58.5	3.7	77.9	1393.7	15.4	48.7	18.8	4.8
	Nonresp.	51	3962.3	31.3	10.9	13.2	39.1	357.8	70.3	25.4	102.5	44.6	2.1	45.9	952.9	19.8	86.3	31.8	10.9

Values are the median values of the particular subgroups in pg/ml; statistically significant factors identified by the Mann-Whitney-*U*-Test/Kruskal-Wallis-Test are marked in bold, statistical significance was assumed as a *p*-value of < 0.05.

**Table 1B:** Association of circulating cytokines and clinicopathological factors

Characteristics		n	IL-1Ra	IL-6	IL-12	IL-17	CCL11	FGF b	GM-CSF	IFN-γ	CXCL-10	MCP-1	MIP-1α	MIP-1β	RANTES	TNF-α	TGF-β1	TGF-β2	TGF-β3
Gender	male	69	2742.0	34.5	10.3	14.7	50.1	430.6	69.9	28.9	168.7	58.5	3.1	48.5	1282.9	15.4	86.3	28.5	9.1
	female	16	8980.3	28.3	14.3	14.7	65.8	283.3	64.6	37.3	632.3	57.1	3.7	46.6	1017.2	20.9	45.9	18.8	4.8
Localization	AEG I/II	44	3178.5	27.1	11.1	16.1	42.6	324.3	69.3	28.9	148.9	57.1	3.1	53.7	1182.6	13.8	86.3	27.8	7.8
	SCC	34	5410.6	45.8	10.9	14.7	51.0	430.6	70.3	28.9	217.3	58.5	3.7	46.6	1262.7	22.5	72.2	28.0	8.7
Grading	G1/2	32	3856.7	28.3	10.9	14.7	42.6	357.8	71.0	28.9	247.0	58.5	2.3	46.6	1962.6	16.2	73.9	28.0	8.0
	G3/4	38	4414.8	27.1	11.1	14.7	58.2	339.8	66.6	28.9	148.9	49.2	3.1	48.5	918.8	17.0	86.0	27.3	10.8
cT Category	cT1	0																	
	cT2	5	8980.3	139.8	9.8	19.7	13.6	344.3	69.9	28.9	43.0	103.5	2.4	26.5	1917.8	19.4	196.3	43.8	7.7
	cT3	69	3856.7	31.3	11.1	14.7	50.1	353.2	70.1	28.9	203.2	58.5	3.3	48.5	1220.5	17.0	73.9	28.0	8.0
	cT4	4	3388.1	15.8	18.1	18.1	84.7	405.9	66.8	26.2	265.5	29.3	7.7	65.8	1617.8	18.5	101.2	28.9	13.3
cN Category	cN0	17	1840.4	41.9	9.8	14.7	37.8	459.1	69.3	28.9	247.0	69.2	2.3	48.5	1017.2	19.4	72.2	28.5	11.1
	cN+	61	3962.3	25.8	11.3	14.7	53.4	344.3	70.1	28.9	158.0	50.3	3.7	63.2	1262.7	17.0	81.8	27.8	5.9
cM Category	cM0	67	3909.5	32.2	10.7	14.7	46.4	348.8	69.3	30.7	210.2	57.8	2.5	46.3	1241.6	17.2	80.6	28.2	8.7
	cM1	11	2966.4	21.0	19.5	15.0	54.6	348.7	70.0	28.0	113.0	56.3	5.1	80.0	1316.2	11.8	80.7	27.1	5.2
pT Category	pT0	17	3962.3	25.8	10.5	13.2	24.1	357.8	69.3	23.5	75.9	38.1	2.1	24.4	952.9	18.6	21.4	18.1	3.8
	pT1	8	3088.2	127.6	12.5	18.0	87.2	403.5	70.2	27.0	158.9	150.5	5.5	106.4	1518.3	23.6	111.5	37.7	3.7
	pT2	10	5552.4	28.1	10.2	16.9	71.0	375.6	72.2	27.1	230.1	55.7	3.1	44.7	1640.2	15.7	75.5	27.6	13.1
	pT3	38	4135.7	31.1	11.2	15.4	55.8	324.6	65.8	32.4	348.6	61.5	3.8	83.5	1388.2	16.2	87.2	31.1	12.0
	pT4	4	897.2	15.1	8.2	12.5	11.6	273.6	74.1	28.9	25.2	14.9	2.2	19.8	169.7	10.6	86.0	48.2	23.3
pN Category	pN0	36	3585.6	50.1	9.8	14.7	42.6	406.9	70.3	28.9	158.0	72.8	2.3	48.5	1282.9	19.4	81.8	30.4	8.0
	pN+	40	3856.7	24.8	11.3	16.1	53.4	310.7	66.6	32.4	331.5	49.2	3.7	77.9	1182.6	15.4	79.3	27.0	9.4
pM Category	pM0	74	3909.5	30.2	11.0	14.7	50.6	348.7	69.6	28.9	199.3	57.8	3.2	48.5	1241.6	17.2	76.6	28.0	8.0
	pM1	3	2543.5	29.5	14.7	25.6	32.9	535.9	55.0	30.7	266.1	68.0	12.0	60.6	2105.4	11.8	134.8	31.4	16.9
Resection status	R0	68	3585.6	29.1	11.3	14.7	53.4	406.9	70.1	28.9	203.2	58.5	3.1	48.5	1262.7	17.0	81.8	28.0	7.8
	R1/2	10	5595.3	33.0	9.8	14.7	31.9	221.4	65.0	32.4	195.4	38.5	3.9	89.0	584.6	18.6	72.2	30.4	10.8
Response																			
Clinical	Resp.	21	3962.3	28.3	11.3	14.7	39.1	344.3	70.3	25.4	75.9	38.5	2.4	26.5	1118.7	20.9	81.8	27.8	7.7
	Nonresp.	55	3523.7	30.2	10.8	16.3	52.2	365.9	68.9	32.4	244.9	57.8	3.7	51.1	1338.3	15.8	82.8	28.2	10.8
Histopathological	Resp.	26	3190.7	29.1	11.1	16.1	51.0	325.0	69.3	28.9	247.0	58.5	3.7	77.9	1393.7	15.4	48.7	18.8	4.8
	Nonresp.	51	3962.3	31.3	10.9	13.2	39.1	357.8	70.3	25.4	102.5	44.6	2.1	45.9	952.9	19.8	86.3	31.8	10.9
Characteristics		n	IL-1Ra	IL-6	IL-12	IL-17	CCL11	FGF b	GM-CSF	IFN-γ	CXCL-10	MCP-1	MIP-1α	MIP-1β	RANTES	TNF-α	TGF-β1	TGF-β2	TGF-β3
Gender	male	69	170.8	14.3	30.9	273.8	118.1	64.7	8.4	153.7	790.1	87.8	5.9	114.8	15994.2	52.7	32095.8	791.1	307.3
	female	16	269.6	12.9	54.3	260.3	84.5	68.0	11.4	186.1	1107.1	94.0	6.4	117.5	18804.3	63.0	30625.4	787.4	296.5
Localisation	AEG I/II	44	162.5	13.0	26.2	258.2	105.6	64.7	8.4	145.4	812.2	89.9	5.5	115.0	15585.8	47.1	29746.7	772.9	305.1
	SCC	34	225.7	15.6	53.7	336.6	118.8	78.9	12.4	186.1	843.1	90.6	7.3	111.5	18507.6	63.0	33125.4	807.4	314.2
Grading	G1/2	32	183.1	13.9	30.9	274.3	111.8	74.3	12.4	155.1	793.1	94.0	6.4	118.6	17889.5	53.6	31914.3	801.9	305.1
	G3/4	38	203.5	19.9	33.3	260.8	104.1	65.5	8.4	164.6	984.2	93.5	5.6	113.6	15306.1	56.4	29828.9	791.1	305.1
cT Category	cT1	0																	
	cT2	5	170.8	20.6	33.3	266.0	101.1	50.2	6.7	164.6	699.7	79.2	5.2	84.4	16064.7	60.2	32009.2	816.4	315.9
	cT3	69	195.4	13.6	30.9	273.8	113.1	68.0	8.4	156.4	812.2	89.9	5.9	114.4	16431.4	52.7	29883.8	776.5	305.6
	cT4	4	203.5	16.1	47.0	306.1	174.2	94.4	38.0	148.2	1876.2	166.7	6.8	189.8	26612.7	43.4	39848.6	1000.0	366.6
cN Category	cN0	17	162.5	10.4	25.7	220.8	114.5	56.3	2.4	145.4	958.0	71.2	4.6	114.7	15901.6	45.3	32341.2	776.5	310.7
	cN+	61	203.5	14.9	34.4	274.7	113.1	75.9	9.7	180.8	796.0	94.5	6.6	114.8	16653.7	60.2	31073.9	796.5	306.2
cM Category	cM0	67	195.4	13.6	31.5	272.5	105.1	66.4	8.4	160.5	817.5	88.8	5.9	117.7	16940.6	52.7	31557.0	782.0	307.3
	cM1	11	203.5	18.2	46.4	261.7	136.5	68.0	8.8	153.7	984.2	93.5	6.6	94.3	15667.4	56.4	31386.7	810.1	298.8
pT Category	pT0	17	219.7	11.8	51.3	317.4	119.5	69.6	10.1	170.1	981.6	86.7	5.7	101.2	18608.5	52.7	33125.4	774.7	307.3
	pT1	8	195.4	14.3	29.7	273.8	104.6	52.1	8.8	175.4	764.6	81.4	6.6	120.3	15667.4	63.9	32424.9	808.3	305.6
	pT2	10	285.3	21.7	38.0	288.2	111.3	72.3	10.3	194.5	963.5	97.8	8.6	123.0	19124.6	67.2	33838.7	822.7	323.3
	pT3	38	195.4	14.9	30.3	256.4	119.0	66.4	8.8	142.6	867.1	93.5	5.6	115.0	16653.7	51.8	29883.8	780.2	302.8
	pT4	4	162.5	13.0	44.6	335.7	77.5	77.4	2.4	167.4	748.5	89.9	6.7	81.3	16516.7	60.2	24488.7	727.0	314.2
pN Category	pN0	36	170.8	11.8	44.6	273.8	122.5	64.7	4.7	156.4	863.3	86.7	5.7	115.0	16653.7	52.7	32944.5	800.1	307.3
	pN+	40	199.4	15.5	29.4	268.6	106.1	68.8	13.1	161.9	817.5	94.8	6.2	114.2	16474.1	57.4	29761.4	773.8	303.9
pM Category	pM0	74	195.4	13.6	32.1	271.2	113.6	66.4	8.8	164.6	822.8	87.8	5.9	114.8	16653.7	54.6	32095.8	791.1	306.2
	pM1	3	203.5	19.9	53.7	306.1	104.1	94.4	13.9	148.2	1607.1	98.6	6.8	109.9	16516.7	52.7	29450.3	778.3	314.2
Resection status	R0	68	195.4	13.6	32.1	266.9	113.6	65.5	8.8	153.7	889.8	87.8	5.7	113.6	16431.4	52.7	29938.6	778.3	305.1
	R1/2	10	199.4	15.7	43.4	309.6	103.3	80.0	2.5	212.3	694.6	104.7	6.7	147.1	18013.5	66.7	35902.0	858.5	328.4
Response																			
Clinical	Resp.	21	211.6	14.9	46.4	300.0	95.4	69.6	8.8	156.4	889.8	89.9	5.6	107.5	15809.1	50.8	30390.6	766.5	307.3
	Nonresp.	55	195.4	13.3	31.5	270.8	119.3	65.9	8.4	155.1	837.7	93.5	5.9	121.4	17418.9	52.7	32271.7	807.4	306.2
Histopathological	Resp.	26	170.8	14.3	30.3	260.8	105.6	66.4	8.4	153.7	768.9	89.9	5.9	114.8	16516.7	52.7	32610.6	785.6	307.3
	Nonresp.	51	231.7	12.4	52.5	318.3	119.5	69.6	12.2	170.1	1103.8	87.8	6.1	114.4	17372.2	62.0	30506.2	786.5	306.2

Values are the median values of the particular subgroups in pg/ml; statistically significant factors identified by the Mann-Whitney-*U*-Test/Kruskal-Wallis-Test are marked in bold, statistical significance was assumed as a *p*-value of < 0.05.

### Cytokines and response to chemotherapy

Histopathological responder had higher serum levels of IL-12 and lower levels of CXCL10 than nonresponding patients (*p* = 0.041 and *p* = 0.032). In the tumor tissue histopathological responders had higher levels of TGF-β1 and TGF-β2 (*p* = 0.033, *p* = 0.007).

Looking separately at patients with AEG I/II and SCC, in both subgroups histopathological responders had higher levels of TGF-ß2 (*p* = 0.049 and *p* = 0.043), Supplementary Table 2.

### Cytokines and prognostic impact

**Figure 1:**
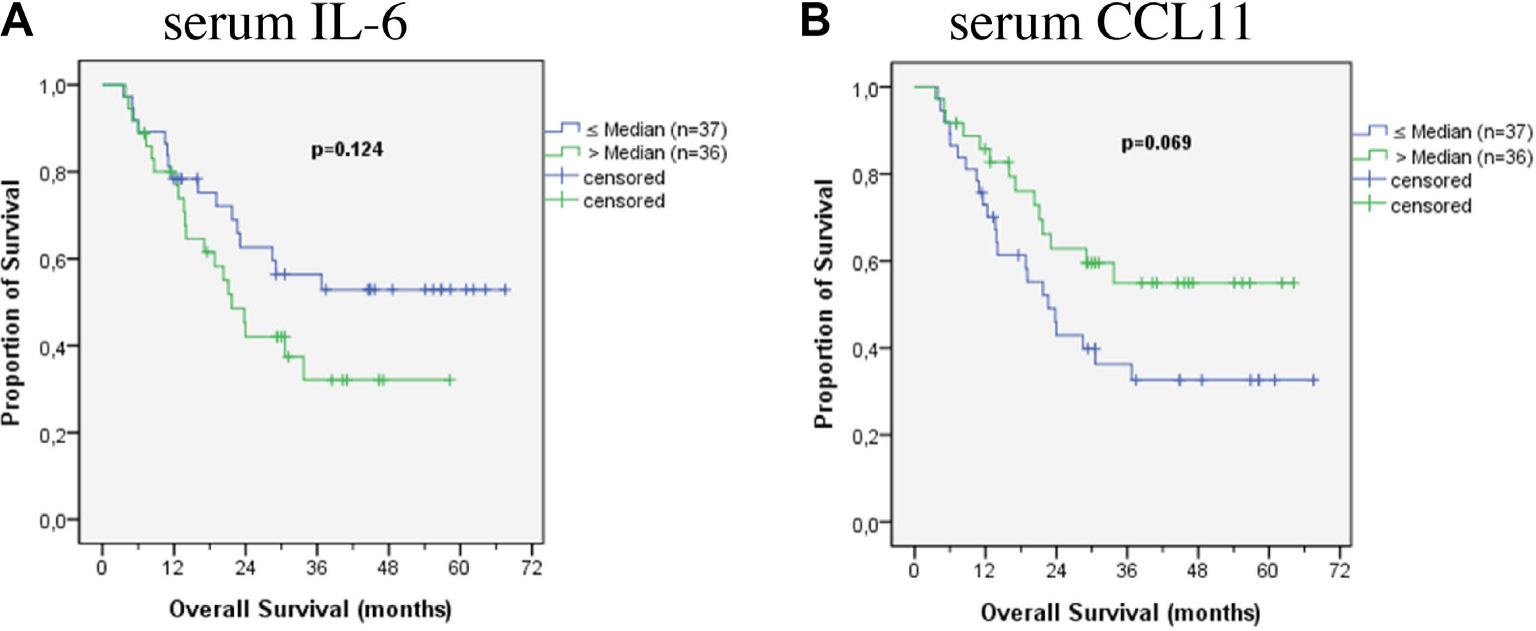
Survival according to cytokines in patients’ serum

**Figure 2:**
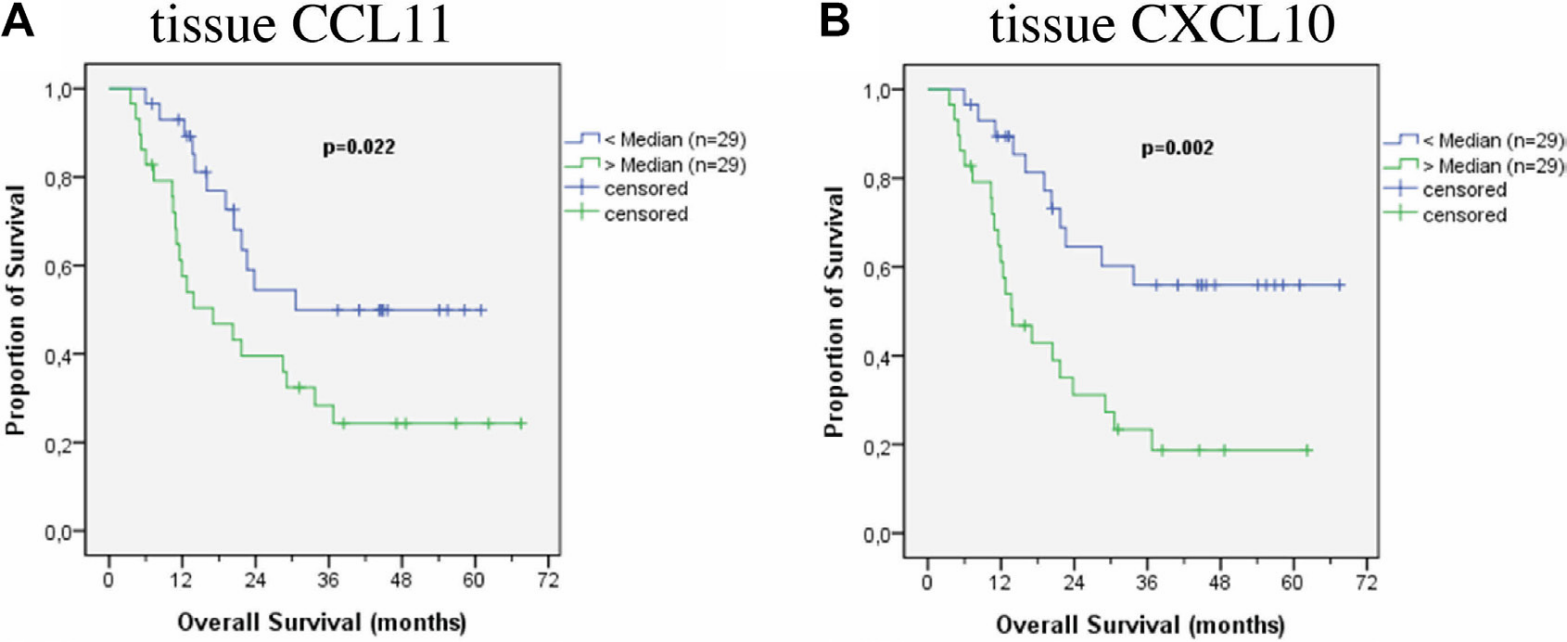
Survival according to cytokines in patients’ tumor tissue

To compare cytokine levels with regard to their prognostic impact, the median was used as a cut-off. No factors were found to have prognostic relevance in the patients’ serum, though high IL-6 levels showed a trend to be associated with worse prognosis (*p* = 0.124) (Figure 1A). IL-6 levels higher than 50 pg/ml were significantly associated with adverse prognosis (*p* = 0.004). Also CCL11 levels in the serum might have a prognostic impact, even if results are statistically not significant. Patients with CCL11 levels above the median survived longer (*p* = 0.069) (Figure 1B). In the tissue specimen two chemokines were associated with prognosis: lower levels of CCL11 (Figure 2A) and CXCL10 (Figure 2B) were associated with better prognosis (*p* = 0.022 and *p* = 0.002). Median survival of patients with lower levels of CCL11 was 30.6 months (standard deviation not calculated) while median survival in patients with higher levels was 17.1 ± 5.5 months (6.4–27.7 95% CI). Median survival of patients with lower levels of CXCL10 was not reached at time of the study, 13.8 ± 3.1 (7.9 – 19.8 95%CI) months for patients with higher levels. Combining CCL 11 and CXCL 10 defines a patients subgroup with dismal prognosis if both factors are above the median (median survival 11.9 ± 1.4 months (9.2;14.7 95%CI) versus median survival 28.5 ± 6.0 months (16.8;40.2 months 95% CI) if one factor is above the median. Median survival is not reached in the favourable subgroup of patients with both factors below the median (*n* = 22). TGF-β3 showed a trend to be associated with prognosis in patients’ tissue (*p* = 0.102). The median survival and the significant cytokines for all patients are shown in Table 2.

**Table 2A:** Prognostic impact of serum cytokines

Serum factor	Median		Median Survival	95% CI	3-Y-S (%)	*p* Value
IL-1ra	195.4	≤ Median	29.1 ± 7.5	14.5–43.8	41.8%	0.720
		> Median	33.7 ± n.c.	–	45.1%	
IL-6	14.1	≤ Median	n.r.	–	52.9%	0.124
		> Median	21.7 ± 2.5	16.7–26.6	32.1%	
IL-12	32.1	≤ Median	30.6 ± 8.6	13.8–47.4	44.1%	0.998
		> Median	24.0 ± 7.4	9.5–38.4	43.0%	
IL-17	271.2	≤ Median	30.6 ± n.c.	–	45.3%	0.717
		> Median	23.0 ± 7.5	8.4–37.7	42.0%	
CCL11	113.1	≤ Median	22.6 ± 3.4	16.0–29.3	32.6%	0.069
		> Median	n.r.	–	55.0%	
FGF basic	66.4	≤ Median	30.6 ± 8.6	13.7–47.5	44.7%	0.849
		> Median	24.0 ± 7.0	10.2–37.8	41.8%	
GMCSF	8.8	≤ Median	28.5 ± 6.0	16.8–40.2	43.4%	0.732
		> Median	33.7 ± 9.4	15.2–52.2	42.4%	
IFN-γ	156.4	≤ Median	30.6 ± 8.3	14.3–46.9	41.9%	0.689
		> Median	24.0 ± n.c.	–	46.6%	
CXCL-10	822.8	≤ Median	22.6 ± 6.6	9.8–35.5	41.4%	0.502
		> Median	33.7 ± 6.5	21.0–46.4	44.9%	
MCP1 (MCAF)	89.9	≤ Median	24.0 ± 4.5	15.1–32.8	39.6%	0.549
		> Median	36.7 ± n.c.	–	46.8%	
MIP1a	5.9	≤ Median	30.6 ± 8.7	13.5–47.7	43.4%	0.940
		> Median	24.0 ± 8.1	8.1 –39.9	43.5%	
MIP1b	114.8	≤ Median	30.6 ± n.c.	–	47.6%	0.661
		> Median	29.1 ± 7.8	13.9–44.4	39.2%	
Rantes	16516.7	≤ Median	30.6 ± n.c.	–	49.5%	0.366
		> Median	21.7 ± 8.2	5.6–37.9	36.6%	
TNFa	52.7	≤ Median	30.6 ± 5.3	20.2–40.9	42.2%	0.938
		> Median	23.0 ± n.c.	–	45.6%	
TGFβ-1	31557.0	≤ Median	30.6 ± n.c.	–	48.3%	0.737
		> Median	28.5 ± 7.2	14.3–42.7	37.3%	
TGFβ-2	791.1	≤ Median	23.8 ± 5.1	13.9–33.7	40.8%	0.729
		> Median	33.7 ± n.c.	–	45.4%	
TGFβ-3	306.2	≤ Median	n.r.	–	51.7%	0.222
		> Median	21.7 ± 5.6	10.8–32.6	34.5%	

Median survival shown in months; n.r.: not reached; n.c.: not calculated; CI: confidence interval; 3-Y-S: 3-Year-Survival; for reason of small sample size standard deviation and confindence interval could not be calculated for all cases.

**Table 2B:** Prognostic impact of cytokines in the tumor tissue

Tissue factor	Median		Median Survival	95% CI	3-Y-S (%)	*p* Value
IL-1ra	3721.1	< Median	21.7 ± 2.6	16.7–26.8	26.4%	0.488
		> Median	36.7 ± n.c.	–	46.3%	
IL-6	30.2	< Median	29.1 ± 8.9	11.6–46.6	38.8%	0.470
		> Median	21.7 ± 6.0	9.8–33.5	34.7%	
IL-12	11.0	< Median	22.6 ± 2.8	17.1–28.1	29.6%	0.627
		> Median	29.1 ± 9.3	10.9–47.3	44.3%	
IL-17	14.7	< Median	21.7 ± 2.7	16.4–27.1	33.5%	0.840
		> Median	28.5 ± 7.2	14.5–42.5	40.3%	
**CCL11**	50.6	< Median	30.6 ± n.c.	–	49.9%	**0.022**
		> Median	17.1 ± 5.5	6.4–27.7	24.3%	
FGF basic	348.7	< Median	36.7 ± n.c.	–	46.3%	0.197
		> Median	20.5 ± 4.6	11.4–29.5	27.8%	
GMCSF	69.6	< Median	20.5 ± 3.9	12.9–28.1	26.8%	0.279
		> Median	30.6 ± n.c.	–	46.9%	
IFN-γ	28.9	< Median	22.6 ± 2.7	17.3–27.9	32.1%	0.968
		> Median	29.1 ± 8.0	13.4–44.9	42.3%	
**CXCL-10**	199.3	< Median	n.r.	–	55.9%	**0.002**
		> Median	13.8 ± 3.1	7.9–19.8	18.7%	
MCP1(MCAF)	57.8	< Median	29.1 ± 9.0	11.4–46.8	38.3%	0.384
		> Median	21.7 ± 6.8	8.3–35.0	34.8%	
MIP1a	3.2	< Median	28.5 ± 6.9	15.0–42.0	39.2%	0.297
		> Median	21.7 ± 6.9	8.1–35.3	34.3%	
MIP1b	48.5	< Median	22.6 ± 6.2	10.4–34.8	40.0%	0.545
		> Median	21.7 ± 7.5	6.9–36.5	33.6%	
Rantes	1241.6	< Median	28.5 ± 9.3	10.3–46.7	43.6%	0.235
		> Median	21.7 ± 6.1	9.6–33.7	30.8%	
TNFa	17.0	< Median	21.7 ± 6.6	8.9–34.6	33.3%	0.932
		> Median	28.5 ± 8.3	12.1–44.8	41.0%	
TGFβ-1	80.6	< Median	20.5 ± n.c.	–	43.6%	0.773
		> Median	28.5 ± 7.2	14.3–42.7	31.9%	
TGFβ-2	28.0	≤ Median	20.3 ± 5.1	13.9–33.7	37.4%	0.696
		> Median	28.5 ± n.c.	–	36.8%	
TGFβ-3	8.0	≤ Median	n.r.	–	51.4%	0.102
		> Median	20.3 ± 5.6	10.8–32.6	25.0%	

Median survival shown in months; n.r.: not reached; n.c.: not calculated; CI: confidence interval; 3-Y-S: 3-Year-Survival; statistically significant factors are marked in bold; for reason of small sample size standard deviation and confindence interval could not be calculated for all cases.

**Figure 3:**
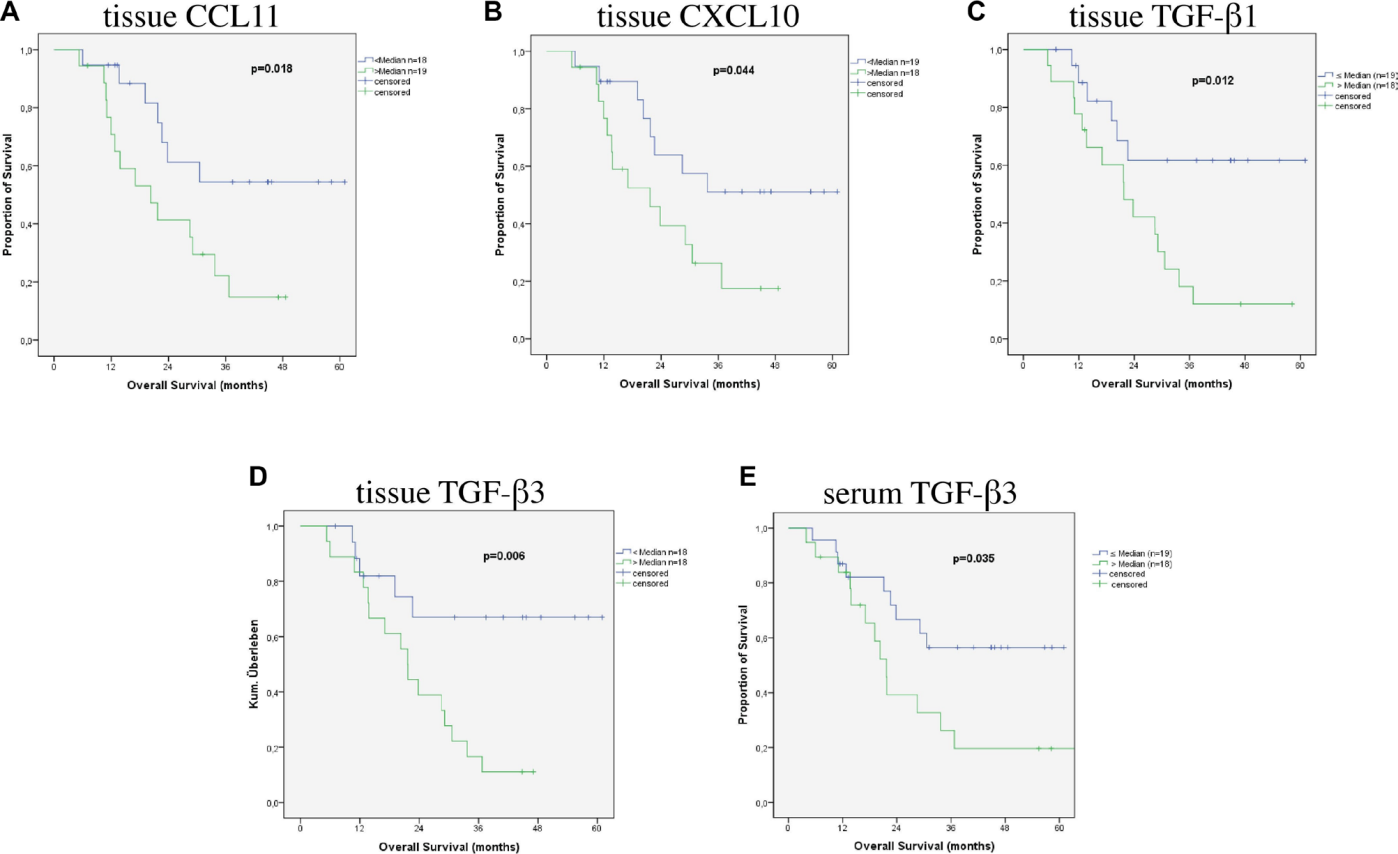
Survival according to cytokines in patients with AEG I/II

In the subgroup of patients with AC (AEG I/II) CCL11 (Figure 3A) and CXCL10 (Figure 3B) in the tissue were associated with patients’ prognosis (*p* = 0.018 and *p* = 0.044) in accordance to the results in all patients. Furthermore an association with prognosis was found for TGF-β1 (Figure 3C) and TGF-β3 (Figure 3D) in the tumor tissue (*p* = 0.012 and *p* = 0.006). Median survival of patients with low tissue levels of TGF-β1 and TGF-β3 was not reached at time of the study, while median survival of patients with higher levels of TGF-β1 was 21.7 ± 4.5 (12.9 – 30.6 95%CI) months and 21.7 ± 1.6 (18.6 – 24.7 95% CI) months for patients with higher tissue levels of TGF-β3. In the serum TGF-β3 (Figure 3E) levels were also found as significant prognostic factor (*p* = 0.035).

**Figure 4:**
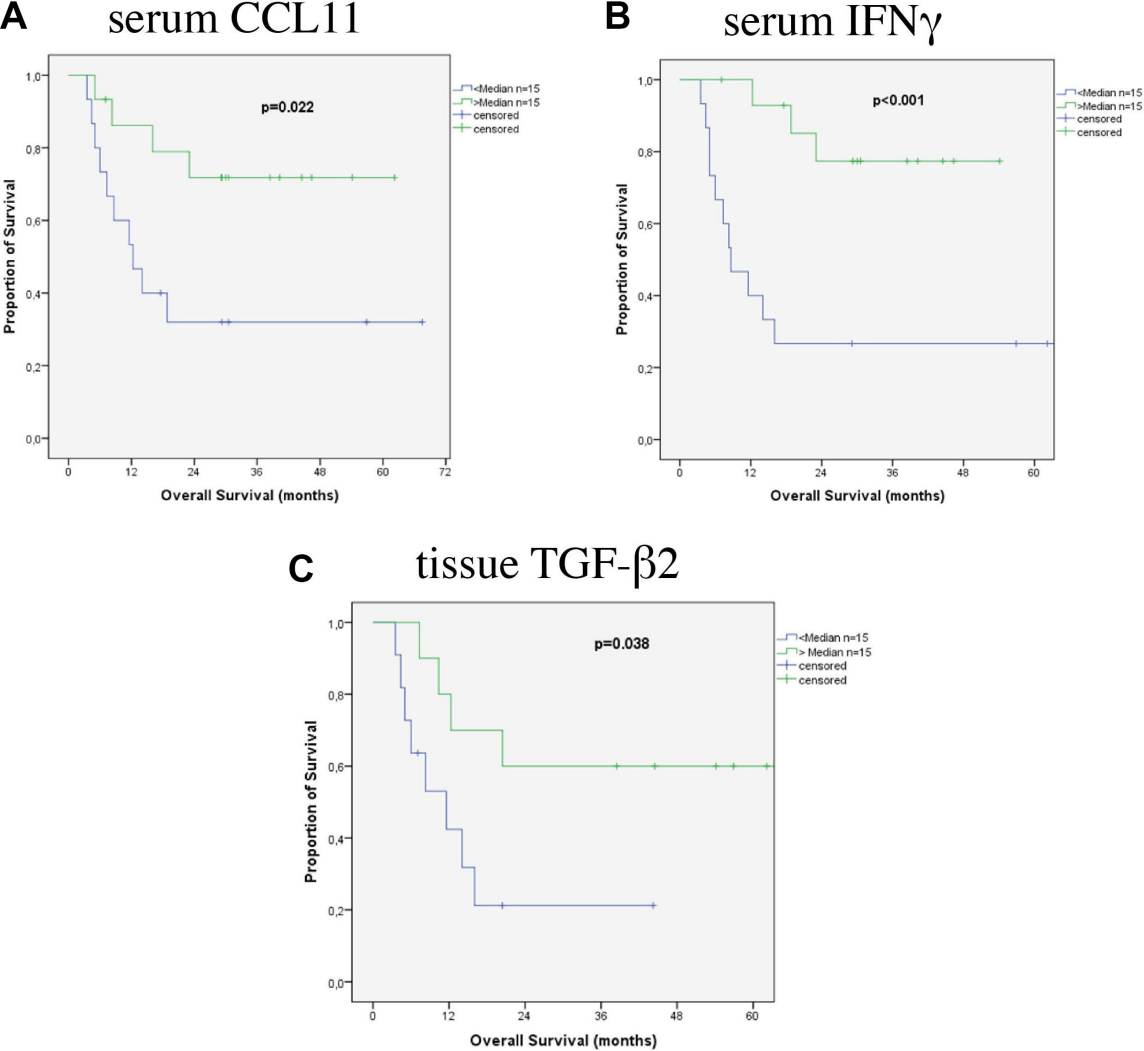
Survival according to cytokines in patients with SCC

In patients with SCC the only relevant prognostic factors in the serum were CCL11 (Figure 4A) and IFNγ (Figure 4B) (*p* = 0.022 and *p* = 0.001). In the tumor tissue TGF-β2 (Figure 4C) was associated with prognosis (*p* = 0.038). Median survival of patients with low tissue levels of TGF-β2 was 11.6 ± 4.1 (3.5 – 19.6 95% CI) months, it was not reached at point of this study in patients with higher levels.

Survival data for the respective subgroups are shown in Supplementary Table 4.

### Receiver operating characteristics (ROC) curves

The median was used as a cut-off for survival analysis. To evaluate this threshold we performed receiver operating characteristics for the statistically significant cytokines (and cytokines with a *p* ≤ 0.1 in Kaplan Meier analysis). By using Youden's index, possible – more appropriate – cut-offs were determined. Receiver operating characteristics and the optimal cut-offs are shown in Table 3.

Table 3Receiver operating characteristics (ROC) for relevant factors

**Table:** 

A: all patients						
Cytokine	Median	Sensitivity/specificity	Best cut-point	Sensitivity/specificity	AUC	Youden Index
CCL11 tissue	50.6	63.6/68	30.0	75.8/56	0.655	0.318
CXCL-10 tissue	199.3	66.7/72	199.3	66.7/72	0.688	0.387
CCL11 Serum	113.1	62.2/61.1	89.4	48.6/77.8	0.619	0.264
TGF ß3 tissue	8.02	72.4/71.4	11.9	62.1/90.5	0.789	0.53

Median/Best cut-point values in pg/ml; AUC: area under the curve.

**Table:** 

B: AEG I/II						
Cytokine	Median	Sensitivity/specificity	Best cut-point	Sensitivity/specificity	AUC	Youden Index
CCL 11 tissue	42.6	66.7/68.9	30.4	81.0/62.5	0.682	0.435
CXCL-10 tissue	148.8	66.7/68.7	113.0	71.4/68.7	0.685	0.401
TGF ß1 serum	29746.7	63.6/65.0	28868.7	77.3/65.0	0.689	0.423
TGF ß1 tissue	86.3	80.0/80.0	87.2	75.0/86.7	0.818	0.617
TGF ß3 serum	305.05	60.0/66.7	288.0	80.0/53.3	0.643	0.333
TGF ß3 tissue	7.85	80.0/86.7	7.85	80.0/86.7	0.860	0.67

Median/Best cut-point values in pg/ml; AUC: area under the curve.

**Table:** 

C: SCC						
Cytokine	Median	Sensitivity/specificity	Best cut-point	Sensitivity/specificity	AUC	Youden Index
CCL11 serum	118.8	71.4/68.7	90.7	64.3/88.0	0.763	0.52
IFN-γ serum	186.1	78.6/68.7	207.1	85.7/68.7	0.732	0.688
TGFß2 serum	807.36	76.9/64.3	809.2	76.7/64.3	0.670	0.412
TGFß2 tissue	28.0	66.7/66.7	28.7	66.7/66.7	0.583	0.334

Median/Best cut-point values in pg/ml; AUC: area under the curve, SCC: squamous cell carcinoma.

As in some factors the optimal cut-off was not near the median, using the optimal cut-off a better sensitivity/specificity for survival was observed and it was used to recalculate the prognostic impact of the different factors.

In all patients the calculated optimal cut-off for CCL 11 in the serum is lower than the median. Using this cut-off survival time between the two groups is significantly different (median survival below the optimal cut-off 18.8 ± 4.2 months, (10.6; 27.0 95% CI) versus median survival not reached above the optimal cut-off, *p* = 0.012). CCL 11 in the tissue is also significant for prognosis, using the optimal cut-off as threshold (*p* = 0.034). Also TGF ß3 in the tissue is calculated as significant prognostic factor using the optimal cut-off. Patients with a TGF ß3 level in the tissue below the cut-off survived longer: median survival not reached versus 17.5 ± 5.1 months (7.0;27.1 95% CI ).

In patients with AC CCL 11 and CXCL 10 in the tissue were also confirmed as prognostic factors with the optimal cut-off (*p* = 0.013, *p* = 0.032). The prognostic impact of TGF ß3 in the tissue with the new cut-off lost statistical significance (*p* = 0.059). In contrast TGF ß1 in the serum gained statistical significance: patients with TGF ß1 in the serum below the optimal cut-off survived longer: median survival not reached versus 21.7 ± 1.1 months (19.6; 23.9 95% CI ), *p* = 0.019.

In patients with SCC the optimal cut-offs differed from the median for CCL 11 and INFγ. Both factors were also confirmed as prognostic factors (*p* = 0.003; *p* = 0.003).

## DISCUSSION

This study investigated 17 cytokines in serum and corresponding tumor tissue of patients with esophageal cancer (AC and SCC) who underwent tumor resection in the Department of Surgery at the University of Heidelberg.

To better understand tumor growth and progression, it is important to understand and consider the tumor microenvironment as a complex network in which cytokines serve as ways to communicate. Most studies reporting cytokine expressions in esophageal cancer are restricted to one or two factors. However, the tumor microenvironment is a complex network in which many different factors orchestrate tumor growth and progression. Strength of this study is the investigation of several factors simultaneously in patients’ serum and the corresponding tumor tissue.

Response to chemotherapy is known and accepted as a relevant prognostic factor in patients with carcinoma of the esophagus, while it is unclear if nonresponding patients have benefit from preoperative systemic treatment [23, 24]. However algorithms to predict response are still not integrated in clinical routine as effective methods are missing. Therefore biomarkers predicting response and prognosis in patients with esophageal cancer are of greatest interest. Response rates mostly range between 20 and 50% and several studies indicate that only patients who respond to neoadjuvant therapy seem to benefit from it [8, 9, 25, 26]. Metabolic response evaluation by for example PET CT has been investigated for adenocarcinoma of the esophagus [27] but was not transferred to clinical routine due to lack of multicenter validation [28].

In this patient series histopathological responders had higher levels of IL-12 and lower levels of CXCL10 in the serum as well as higher level of TGF-β1 and TGF-β2 in the tissue. But as serum and tissue was collected after therapy no definitive conclusion regarding feasibility of response prediction can be made. To answer this important question pretherapeutic cytokine detection would necessary.

Association with other clinicopathologial factors has been reported for several cytokines in esophageal cancer. For IL-6, IL-8, IL-18, IL-19, IL-27, TGF-β, FGFb and PDGF-BB associations with categories of the TNM-classification have been reported [17, 29–35]. In this study we found an association of MIP-1β, TGF-β2 und TGF-β3 in the serum with the cT category and an association of MIP-1α and TNF-α with the cN category. An association of TGFβ with the T category has also been described by Fukai et al. [31]. We found that higher concentrations of IL-6 in the patients’ serum were associated with a positive postoperative lymph node status. These findings are in line with one study that investigated the association in patients with carcinoma of the esophagus [17]. A main limitation of our study is the fact that all samples were taken after neoadjuvant therapy and therefore could be influenced by the treatment. Since this study aimed to identify potential markers for prediction, results have to be validated in a prospective study with comparison of pre- and post-therapeutic evaluation of serum levels.

Even though most cytokine levels did not differ significantly between AC and SCC, different expression of several cytokines may indicate the biological diversity of the two types, as the median concentration of IL-1ra, IL-12, IL-17, FGFb, Mip-1α and Rantes in the serum and TNFα in the tumor tissue differed between the two entities AC and SCC, indicating biological differences. In clinical studies patients with AC and SCC are analysed together as one cohort. The CROSS-trial [36] as one of the first studies showing a benefit for neoadjuvant treatment included patients with AC and SCC. For investigating clinical outcome parameters pooling of patients with AC and SCC is therefore well established. For analysing the prognostic impact of the described cytokines a certain sample size is necessary to identify potential markers. Since in this study a wide spectrum of different markers is tested and to achieve this sample size a pooled analysis of AC and SCC samples was performed even if this may skew the results due to the heterogeneous patient cohort.

In this study when using the median as cut off none of the investigated pro-inflammatory cytokines in the serum were significantly associated with patients’ prognosis. A trend could be seen for IL-6 in the serum, as patients with lower concentrations of this factor had better prognosis than patients with higher concentrations, but this did not reach statistical significance. However, concentrations >50 pg/ml were found as a significant adverse prognostic factor. IL-6 is a dual property cytokine with pro-inflammatory and anti-inflammatory roles and is regarded as a key regulator in human immune regulation and inflammatory reaction [37]. High serum concentrations of IL-6 have been described for several solid tumors such as lung, breast, pancreatic and gastric cancer [38]. Lukaszewicz-Zajac et al. reported high concentrations of serum IL-6 to be associated with more advanced tumor stages and worse prognosis in patients with esophageal cancer [17]. Also Chen et al. described an association of IL-6 in the tumor tissue in patients with SCC with adverse prognosis [19].

During tumor development and growth, chemokines are important regulators of the tumor microenvironment. Secreted by the tumor and surrounding stromal cells they exert influence on the amount of leukocytes in the tumor environment as well as on angiogenesis [39]. As far as esophageal cancer is concerned little is known about the complex network of chemokines, in which sometimes divergent expressions seem to orchestrate tumor progression [21].

Out of the investigated chemokines CCL11 and CXCL10 were relevant prognostic factors in this study. Lower concentrations of CCL11 in the tumor tissue were associated with better prognosis. CCL11 is a chemokine related to chronic inflammation and may take part in tumor-associated inflammation [40, 41]. For several solid tumors elevated levels of CCL11 either in the serum or in tumor tissue have been reported [41–44]. Little is known about CCL11 expression in esophageal cancer. Interestingly, lower CCL11 levels in patients with SCC were associated adverse prognosis in this study.

The adverse prognosis related to CCL11 levels in patients’ serum in this study might be explained by the fact that the expression in serum and tumor tissue is unrelated and highly-dependent on the immunogenic factors. Cho et al. [45] report that low expression levels of CCL11 in colorectal carcinoma tissue are associated with a worse prognosis. Since colorectal carcinoma is known as a non-immunogenic tumor this might be an immune-evading strategy of the tumor. In SCC tumors low levels of CCL11 were associated with better prognosis compared to patients expressing higher levels of CCL11. This better prognosis could be caused by elevated immune response in the tumor tissue leading to a better long-term survival. Tian et al. reported similar findings in patients with glioblastoma [46]. They found that an overexpression of CCL11 in the tumor was significantly associated with a poor overall survival. This might be caused by a CCL11-dependent activation of cell proliferation, tumor migration and invasion. Salcedo et al. described a CCL11-dependent activation of angiogenic pathways leading to higher vascularization of the tumor tissue [47]. The adverse prognostic impact of CCL11 in SCC- and AEG-tumors could be explained by the different immunogenic profile of the different tumor entities. Higher serum levels of CCL11 might induce higher immunogenic response to the tumor tissue leading to better clinical outcome. Different expression profiles depending on the tissue type might be responsible for the adverse prognostic CCL11-levels in the tumor tissue.

Another prognostic relevant chemokine in the tissue specimen was (CXCL10) Interferon-γ-Inducible Protein. In this study higher concentrations of CXCL10 in the tumor tissue were associated with adverse prognosis. CXCL10 is an immunomodulatory chemokine, which is able to recruit monocytes, T and NK cells [48]. For animal models a tumor growth inhibiting effect has been reported (49–51). However, the role of CXCL10 in human solid tumors has been scarcely investigated. Eck et al. described pleiotropic effects in gastric cancer specimen [52]. AC cell lines constitutively express CXCL10 and this expression is gradable by IFNγ and TNFα [53]. Rajkumar et al. reported an elevated expression of CXCL10 mRNA in gastric cancer tissue specimen when compared with normal tissue as well as a drop of serum CXCL10 after surgery [54]. In the AC subgroup CXCL10 was as well associated with patients’ prognosis in this study. In addition histopathological responders had lower CXCL10 serum levels than nonresponders, emphasizing its potential role in esophageal cancer.

In this study TGF-β3 showed a trend to be associated with prognosis in tumor tissue. The prognostic difference was significant using the calculated optimal cut-off and not the median as a cut-off (with better prognosis with low TGF-ß3 concentrations). In the subgroup of AC tumors low serum concentrations of TGF-β3 and TGF-β1 as well as low tissue levels of TGF-β3 and TGF-ß1 were significantly associated with better prognosis. Interestingly, this association was not found in the SCC subgroup. In SCC low concentrations of TGF-β2 in the tumor tissue were associated with adverse prognosis, a same trend could be observed in the serum. Tumor cells often secrete large amounts of TGF-β. Raised TGF-β1 serum levels have been described for several solid tumors such as gastric cancer, adenocarcinoma of the lung and breast cancer [38]. TGF-ß has an important role in mediating inflammation in gastric cancer. Helicobacter pylori-associated inflammation is mainly induced by TGF-ß [55]. In addition, TGF-ß plays an important role in epithelial-mesenchymal transition of gastric cancer cells [56]. TGF-β is also known for its function to induce an environment of immunotolerance and to inhibit immune activities against the tumor [38]. These functions seem to mainly apply for advanced tumors, while TGF-β has been reported to exert growth inhibiting effects during genesis of the tumor [57]. Several studies described an overexpression of TGF-β1 in the serum or tumor tissue when compared to adjacent tissue or serum of healthy controls for SCC [31, 58, 59].

In this study we evaluated serum and tissue levels of different relevant cytokines and chemokines in neoadjuvantly treated patients. As we found no significant association of circulating cytokines and chemokines with survival in the serum of all patients, changes in cytokine levels during the neoadjuvant therapy have to be considered. Still IL-6 seems to be a relevant factor as high concentrations in the serum (> 50 pg/ml) were found to be a significant adverse prognostic factor. Possibly this limit would be lower in untreated patients. Other studies mostly investigated serum or tissue specimen in untreated patients. Further studies could illuminate the possible changes of circulating cytokine levels in patients with esophageal cancer. Cytokine levels should not only be detected in both serum and tumor tissue, but as well in a sequential way, that is before any kind of therapy, pre- and postoperatively. Additional studies with more patients could help to further unravel the complex cytokine network that forms the tumor microenvironment.

## MATERIALS AND METHODS

### Patient characteristics

As published previously [22], this retrospective study included 78 patients with esophageal squamous cell cancer (*n* = 34) and adenocarcinoma of the gastroesophageal junction (AEG I/II) (*n* = 44). All patients gave written informed consent and the study protocol was approved by the Ethical Committee of the University of Heidelberg. Patients underwent neoadjuvant chemo(radio)therapy followed by resection or explorative operation according to the current guidelines in the Department of Surgery, University of Heidelberg, Germany from 2007 to 2012. The neoadjuvant therapy was mostly applied as an outpatient treatment by the patients’ oncologist.

Most patients with AC received EOX or alternatively FLOT chemotherapy regimens. Patients with SCC principally received a chemoradiotherapy consisting of 36–70 Gy, Cisplatin and 5-Fluoruracil. For further details on neoadjuvant treatment regimens see as well Supplementary Table 3A (as published previously [22]).

A CT scan and an endoscopy were performed as preoperative staging. In the CT scan a decline of more than 50% in wall thickness and a decrease of tumor mass in endoscopy defined patients as clinical responders.

Surgical resection mainly comprised of abdominothoracic en bloc esophagectomy with gastric tube pull-up (Ivor-Lewis procedure). In case of AEG II tumors a transhiatal gastrectomy was performed. Operative procedures are displayed in Supplementary Table 3B [22].

Follow up mostly took place in the National Center for Tumor Diseases, Heidelberg. Patients receiving their follow-up visits in other centers were contacted by phone. Median follow-up of the surviving patients was 40.62 months, while one patient is lost-to-follow-up.

### Histopathological analysis

Histopathological analysis of the pathological specimens took place in the department of pathology in Heidelberg. For histopathological staging TNM classification, R-category and tumor regression grade (TRG) were evaluated. 7th edition of the TNM staging was applied to re-classify patients having been treated before 2010. Becker regression score was used to define TRG [23, 60]. Patients with regression grades 1a and 1b were defined as histopathological responders, patients with grades 2 and 3 as non-responders.

### Blood and tissue sampling and preparation

The method of blood and tissue sampling, preparation and cytokine detection was published previously [22]. The day before surgical resection blood samples were taken by peripheral vein punction or from a central venous catheter and collected in serum tubes. To prevent dilution with blocking saline the first 5ml of the taken blood were discarded when blood was drawn from a central venous catheter. Tubes were then centrifugated at 2.500 g for 10 minutes to extract the serum. Until analysis serum was then stored at −80°. Immediately before analysis serum was diluted 1:4 with a sample diluent.

Directly after the surgical resection tumor tissue specimen were collected and stored at −80°C. By using a cryotome the tissue specimens were cut into sections of 10μm to prepare them for analysis. These sections were then given into a lysis buffer. The concentration of the lysated tissue samples was adjusted to 600 μg/ml.

### Cytokine detection

We detected serum and tissue concentrations of IL-1Ra, IL-6, IL-12, IL-17, CCL 11 (Eotaxin-1), FGF basic, GM-CSF, IFNγ, CXCL10 (IP-10), MCP-1(MCAF), MIP-1α, MIP-1β, Rantes (CCL5), TNFα, TGFβ-1, -2, and -3. Cytokine levels were measured using the BioRad Bio-Plex Pro Human Cytokine 27-Plex Assay and the BioRad Bio-Plex Pro TGFβ 3-plex (Bio-Rad Laboratories, Inc., Hercules, CA 94547, USA) and Luminex two-laser array reader (Bioplex200). Bioplex Manager 6.1. (Bio-Rad Laboratories, Inc., Hercules, CA 94547, USA) was used to acquire standard curves and concentrations.

### Statistical analysis

Continuous variables are shown as median. Mann-Whitney *U* test or Kruskal-Wallis-Test were applied to compare differences in the calculated medians. Categorical data are presented in absolute and relative frequencies. Chi-square-test was used for comparison. To compare cytokine levels we used the median as a cut-off.

Overall survival was detected from point of diagnosis until death. Survival analysis was performed using the Kaplan-Meier method. Log-rank test was applied for differences in survival time. Statistical significance was assumed as a *p*-value of < 0.05.

Receiver operating characteristics (ROC) including the area under the curve (AUC) were used to calculate the best cut-off of the respective cytokine concentrations using the status (alive/deceased) as a reference. With the Youden's index (sensitivity+specificity-1) the best cut-point was calculated.

For all statistical analyses we used SPSS software version 20.0 (SPSS, Inc., Chicago, Illinois, USA).

## CONCLUSIONS

Cytokines as part of the tumor microenvironment influence tumor growth and progression. In this study two chemokines (CCL11 and CXCL10) in the posttherapeutic tumor tissue were associated with prognosis in patients with esophageal cancer. Also TGF-β may be important, especially in patients with AC and was associated with response and prognosis in this study. Also IL-6 seems to play a role in tumor progression and prognosis in patients with carcinoma of the esophagus.

Results are promising but further studies including sequential measurements of cytokine levels are necessary to illuminate their complex network of the tumor microenvironment.

## SUPPLEMENTARY MATERIALS FIGURES AND TABLES





## Abbreviations

SCC: esophageal squamous cell cancer
AC: adenocarcinoma
IL: interleukin
TNF: tumor necrosis factor
TGF: tumor growth factor
AEG: adenocarcinoma of the esophagogastric junction
ROC: Receiver Operating Characteristics
PET: positron emission tomography
CT: computed tomography
AUC: area under the curve.
