# Supplementary material for: Inflammatory cytokines are associated with response and prognosis in patients with esophageal cancer
Source: Oncotarget. 2017 May 7;8(29):47518–32. doi: 10.18632/oncotarget.17671 (PMC5564583; doi:10.18632/oncotarget.17671)
Supplement: Supplementary file 1 [file oncotarget-08-47518-s001.pdf]

Inflammatory cytokines are associated with response and prognosis in patients with esophageal cancer

Supplementary Materials

Supplementary Table 1A: Pretherapeutic patients' characteristics

Characteristics		*n*	%	Median Survival	95% CI	3-Y-S (%)	*p* Value
Total, n		78	100,0%	28,5		42,7%	
Age (years)	63 ± 7,8 (41-80)						
Gender	Male	62	79,5%	28,5 ± 6,3	16,1 - 40,9	42,6%	0,711
	Female	16	20,5%	29,1 ± n.r.	-	45,6%	
Localisation	AEG I/II	44	56,4%	28,5 ± 4,8	19,2 - 37,8	37,7%	0,904
	SCC	34	43,6%	n.r.	-	50,9%	
cT Category	cT1	0					
	cT2	5	6,4%	n.r.	-	60,0%	0,812
	cT3	69	88,5%	23,8 ± 4,0	15,9 - 31,7	42,8%	
	cT4	4	5,1%	36,7 ± 2,5	31,9 - 41,5	33,3%	
cN Category	cN0	17	21,8%	30,6 ± 7,7	15,5 - 45,7	37,8%	0,906
	cN+	61	78,2%	24,0 ± 7,6	9,1 - 38,8	44,6%	
cM Category	cM0	67	85,9%	23,8 ± 4,4	15,1 - 32,5	40,2%	0,317
	cM1	11	14,1%	n.r.	-	57,3%	
Grading	G 1/2	32	41,0%	23,1 ± 4,9	13,4 - 32,7	34,0%	0,428
	G 3/4	38	48,7%	29,1 ± n.r.	-	45,5%	
Lauren initial (AEG)	Intestinal	26	59,1%	30,6 ± 7,7	15,6 - 45,6	40,4%	0,620
	Nonintestinal	13	29,5%	24,0 ± 2,4	19,3 - 28,6	33,8%	

Median Survival shown in months; n.r.: not reached; CI: confidence interval; 3-Y-S: 3-Year-Survival.

Supplementary Table 1B: Postoperative patients' characteristics

Characteristics		*n*	%	Median Survival	95%CI	3-Y-S (%)	*p* Value
pT Category	pT0	17	21,8%	n.r.	-	54,1%	0,464
	pT1	8	10,3%	n.r.	-	72,9%	
	pT2	10	12,8%	n.r.	-	58,3%	
	pT3	38	48,7%	23,8 ± 4,9	14,3 - 33,4	33,8%	
	pT4	4	5,1%	10,4 ± 7,6	0,0 - 25,2	25,0%	
pN Category	pN0	36	46,2%	n.r.	-	66,1%	0,001
	pN+	40	51,3%	20,3 ± 2,7	14,9 - 25,6	21,8%	
pM Category	pM0	74	94,9%	n.r.	-	46,1%	0,235
	pM1	3	3,8%	21,8 ± 21,8	0,0 - 73,3	0,0%	
Grading postop.	G1/2	15	19,2%	22,6 ± n.r.	-	48,1%	0,927
	G3/4	33	42,3%	28,5 ± 6,6	15,5 - 41,4	40,7%	
Lauren postop. (AEG)	Intestinal	22	50,0%	24,0 ± 4,8	14,6 - 33,3	27,8%	0,796
	Nonintestinal	14	31,8%	21,1 ± 2,8	15,7 - 26,6	31,2%	
R Status	R0	68	87,2%	29,1 ± 6,8	15,8 - 42,4	42,7%	0,617
	R1	10	12,8%	19,1 ± 5,2	8,9 - 29,3	40,0%	

Median Survival shown in months; n.r.: not reached; CI: confidence interval; 3-Y-S: 3-Year-Survival.

Supplemental Table 2: Association of cytokines with clinical response and TRG in AEGI/II and SCC

**AEGI/ II**		**IL-1Ra**	**IL-6**	**IL-12**	**IL-17**	**CCL11**	**FGF b**	**GM-CSF**	**IFN-γ**	**CXCL-10**	**MCP-1**	**MIP-1α**	**MIP-1β**	**RANTES**	**TNF-α**	**TGF-β1**	**TGF-β2**	**TGF-β3**
Serum																		
Clinical	Responder	219.1	14.6	39.8	259.9	90.8	66.3	5.6	155.1	1035.5	80.7	5.0	93.3	15274.9	49.9	25640.8	732.5	296.5
	Nonresponder	158.4	12.1	23.6	257.7	112.8	64.7	7.5	139.8	839.7	97.3	5.6	121.9	16029.5	47.1	29828.9	791.1	305.1
TRG	Responder	145.9	9.6	28.6	274.7	113.6	57.7	11.4	131.3	1306.4	109.2	4.5	122.5	15994.2	41.5	29799.5	738.0	305.1
	Nonresponder	162.5	13.3	25.4	257.3	105.1	64.7	7.5	146.8	782.4	88.3	5.6	114.9	15443.0	52.7	29693.8	778.3	305.1
Tissue																		
Clinical	Responder	2590.9	63.5	13.7	12.5	39.1	344.3	74.1	28.9	75.9	188.8	4.3	82.1	1118.7	18.6	86.0	27.8	7.7
	Nonresponder	3190.7	23.9	10.5	16.5	53.4	316.5	66.6	32.4	242.9	49.2	2.7	48.5	1282.9	13.8	881.4	284.9	111.0
TRG	Responder	4951.0	50.1	13.7	16.5	39.1	353.2	73.3	32.4	75.9	149.2	3.3	63.2	652.0	16.2	48.7	**18.1**	3.8
	Nonresponder	2960.3	24.0	10.7	15.0	48.0	320.4	65.6	28.9	219.1	53.7	2.9	45.9	1232.8	13.4	87.2	**31.6**	9.4
SCC																		
Serum																		
Clinical	Responder	211.6	16.1	47.0	318.3	95.4	75.9	10.1	164.6	889.8	96.1	5.9	114.4	17386.8	50.8	32935.9	782.9	311.3
	Nonresponder	289.4	15.8	57.4	346.2	121.0	81.6	12.4	208.3	826.7	90.6	8.9	113.8	19925.5	69.5	33929.2	818.2	315.9
TRG	Responder	318.9	13.0	70.5	336.6	122.2	72.7	16.3	186.1	995.6	87.2	7.7	111.5	17997.7	65.8	33125.4	796.5	307.3
	Nonresponder	203.5	16.1	47.0	354.9	118.1	94.4	12.6	212.3	685.7	93.5	7.9	105.3	19979.0	58.3	35528.3	811.0	321.0
**Tissue**																		
Clinical	Responder	6471.3	20.0	10.3	14.7	46.3	348.8	67.7	21.6	100.5	27.7	**1.5**	**20.2**	1118.9	25.9	72.3	24.1	6.8
	Nonresponder	5410.6	59.3	13.3	14.7	51.0	471.8	73.9	37.3	247.0	81.3	**9.6**	**90.4**	1767.3	18.6	72.2	28.0	10.8
TRG	Responder	3773.9	20.0	10.3	12.5	46.3	414.8	67.6	21.6	163.4	27.7	1.8	22.4	985.1	21.7	41.1	**19.8**	4.8
	Nonresponder	8365.7	59.3	11.3	17.6	51.0	430.6	73.9	38.9	429.0	72.8	13.4	129.6	1962.6	24.0	79.3	37.6	11.1

Values are the median values of the particular subgroups in pg/ml; statistically significant factors identified by the Mann-Whitney-*U*-Test/Kruskal-Wallis-Test are marked in bold, statistical significance was assumed as a *p*-value of < 0.05.

Supplemental Table 3A: Neoadjuvant treatment regimens

Regimen	*n*	%
Chemoradiotherapy	39	50
EOX	26	33.4
FLO(T)	4	5.1
Others	9	11.5

EOX: epirubicin, oxaliplatinum, capecitabine;
FLO: oxaliplatinum, 5-fluorouracil, folinic acid;
FLOT: docetaxel, oxaliplatinum, 5-fluorouracil, folinic acid

Supplemental Table 3B: Operative procedures

Type of Surgery	*n*	%
Abdominothoracic esophagectomy	62	79.5%
Transhiatal extended gastrectomy	13	16.7%
Transmediastinal esophagectomy	2	2.6%
Exploration	1	1.3%

Supplemental Table 4 A: Prognostic value of cytokines in patients with AEGI/II. See_Supplementary_ Table 4A

Supplemental Table 4 B: Prognostic value of cytokines in patients with SCC. See_Supplementary_ Table 4B
