# Supplementary material for: Inflammatory cytokines are associated with response and prognosis in patients with esophageal cancer
Source: Oncotarget. 2017 May 7;8(29):47518–32. doi: 10.18632/oncotarget.17671 (PMC5564583; doi:10.18632/oncotarget.17671)
Supplement: Supplementary file 2 [file oncotarget-08-47518-s002.docx]

**Supplementary Table 4:**

**A) Prognostic value of cytokines in patients with AEGI/II**

| **Serum factor AEGI/II** | **Median** |  | **Median Survival** | **95% CI** | **3-Y-S (%)** | **p Value** |
| --- | --- | --- | --- | --- | --- | --- |
| IL-1ra | 162.5 | ≤ Median | 36.7 ± n.r. | n.r. | 46.8% | p=0.295 |
|  |  | > Median | 21.7 ± 1.8 | 18.3 - 25.2 | 29.1% |  |
| IL-6 | 13.0 | ≤ Median | 36.7 ± n.r. | n.r. | 49.1% | p=0.209 |
|  |  | > Median | 23.8 ± 1.9 | 20.0 - 27.6 | 27.5% |  |
| IL-12 | 26.2 | ≤ Median | 30.6 ± n.r. | n.r. | 46.8% | p=0.472 |
|  |  | > Median | 21.7 ± 1.9 | 18.0 - 25.5 | 28.0% |  |
| IL-17 | 258.2 | ≤ Median | 30.6 ± 5.7 | 19.4 - 41.8 | 40.4% | p=0.696 |
|  |  | > Median | 21.7 ± 2.5 | 16.9 - 26.6 | 36.2% |  |
| CCL11 | 105.6 | ≤ Median | 28.5 ±4.9 | 18.8 - 38.2 | 35.8% | p=0.693 |
|  |  | > Median | 29.1 ± 7.7 | 14.1 - 44.1 | 41.6% |  |
| FGF basic | 64.7 | ≤ Median | 36.7 ± n.r. | n.r. | 46.8% | p=0.337 |
|  |  | > Median | 21.7 ± 1.9 | 18.1 - 25.4 | 29.1% |  |
| GM-CSF | 8.4 | ≤ Median | 29.1 ± 5.9 | 17.5 - 40.7 | 44.0% | p=0.621 |
|  |  | > Median | 24.0 ± 7.3 | 9.7 - 38.3 | 30.3% |  |
| IFN-γ | 145.4 | ≤ Median | 36.7 ± n.r. | n.r. | 48.9% | p=0.230 |
|  |  | > Median | 21.7 ± 1.9 | 17.9 - 25.5 | 28.5% |  |
| CXCL-10 | 812.2 | ≤ Median | 22.6 ± 7.0 | 8.8 - 36.4 | 40.4% | p=0.807 |
|  |  | > Median | 29.1 ± 6.7 | 16.0 - 42.2 | 36.2% |  |
| MCP-1(MCAF) | 89.9 | ≤ Median | 29.1 ± 4.7 | 19.9 - 38.4 | 42.9% | p=0.489 |
|  |  | > Median | 23.8 ± 8.1 | 8.0 - 39.6 | 33.3% |  |
| MIP1a | 5.5 | ≤ Median | 36.7 ± n.r. | n.r. | 48.3% | p=0.159 |
|  |  | > Median | 21.7 ± 1.5 | 18.8 - 24.7 | 24.5% |  |
| MIP1b | 115.0 | ≤ Median | 30.6 ± n.r. | n.r. | 46.0% | p=0.418 |
|  |  | > Median | 23.8 ± 5.3 | 13.4 - 34.2 | 30.7% |  |
| Rantes | 15585.8 | ≤ Median | 28.5 ± 4.8 | 19.1 - 37.8 | 43.5% | p=0.625 |
|  |  | > Median | 29.1 ± 8.2 | 13.0 - 45.2 | 34.4% |  |
| TNFα | 47.1 | ≤ Median | 33.7 ± 5.9 | 22.2 - 45.2 | 42.5% | p=0.453 |
|  |  | > Median | 21.7 ± 2.4 | 17.1 - 26.4 | 32.7% |  |
| TGFβ-1 | 29746.7 | < Median | n.r. | - | 58.1% | p=0.088 |
|  |  | > Median | 21.7 ± 1.0 | 19.7 - 23.7 | 22.7% |  |
| TGFβ-2 | 772.9 | < Median | n.r. | - | 51.7% | p=0.256 |
|  |  | > Median | 28.5 ± 6.1 | 16.6 - 40.4 | 26.8% |  |
| **TGFβ-3** | **305.05** | ≤ Median | n.r. | - | 56.5% | **p=0.035** |
|  |  | > Median | 21.7 ± 1.7 | 18.4 - 25.0 | 19.6% |  |
|  |  |  |  |  |  |  |
|  |  |  |  |  |  |  |
| **Tissue factor AEG I/II** | **Median** |  | **Median Survival** | **95% CI** | **3-Y-S (%)** | **p Value** |
| IL-1ra | 3178.5 | ≤ Median | 23.8 ± 4.4 | 15.2 - 32.4 | 25.9% | p=0.303 |
|  |  | > Median | 36.7 ± 12.3 | 12.6 - 60.9 | 43.8% |  |
| IL-6 | 27.1 | ≤ Median | 33.7 ± 11.0 | 12.1 - 55.3 | 36.7% | p=0.677 |
|  |  | > Median | 28.5 ± 4.8 | 19.2 - 37.8 | 32.5% |  |
| IL-12 (p70) | 11.1 | ≤ Median | 22.6 ± 3.2 | 16.4 - 28.9 | 24.0% | p=0.245 |
|  |  | > Median | 33.7 ± n.r. | - | 48.0% |  |
| IL-17 | 16.1 | ≤ Median | 23.8 ± 2.0 | 19.8 - 27.8 | 30.4% | p=0.869 |
|  |  | > Median | 30.6 ± 7.5 | 15.8 - 45.3 | 39.7% |  |
| **CCL11** | 42.6 | ≤ Median | n.r. | - | 54.4% | **p=0.018** |
|  |  | > Median | 20.3 ± 5.4 | 9.8 - 30.8 | 14.8% |  |
| FGF basic | 324.3 | ≤ Median | 36.7 ±7.0 | 23.0 - 50.5 | 43.9% | p=0.190 |
|  |  | > Median | 22.6 ± 4.3 | 14.2 - 31.1 | 26.0% |  |
| GM-CSF | 69.3 | ≤ Median | 28.5 ± 5.9 | 16.9 - 40.1 | 28.2% | p=0.628 |
|  |  | > Median | 22.6 ± 8.2 | 6.5 - 38.8 | 41.6% |  |
| IFN-γ | 28.9 | ≤ Median | 23.8 ± 4.6 | 14.8 - 32.8 | 29.7% | p=0.834 |
|  |  | > Median | 29.1 ± 8.1 | 13.2 - 45.0 | 42.2% |  |
| **CXCL-10** | 148.9 | ≤ Median | n.r. | - | 51.1% | **p=0.044** |
|  |  | > Median | 21.7 ± 6.3 | 9.4 - 33.9 | 17.5% |  |
| MCP-1(MCAF) | 57.1 | ≤ Median | 33.7 ± 10.4 | 13.4 - 54.0 | 43.2% | p=0.435 |
|  |  | > Median | 23.8 ± 7.2 | 9.7 - 37.9 | 32.9% |  |
| MIP1a | 3.1 | ≤ Median | 30.6 ± 7.8 | 15.3 - 45.9 | 38.2% | p=0.424 |
|  |  | > Median | 23.8 ± 7.7 | 8.8 - 38.8 | 31.3% |  |
| MIP1b | 53.7 | ≤ Median | 30.6 ± 7.8 | 15.3 - 45.9 | 38.2% | p=0.424 |
|  |  | > Median | 23.8 ± 7.7 | 8.8 - 38.8 | 31.3% |  |
| Rantes | 1182.6 | ≤ Median | 33.7 ± 10.9 | 12.4 - 55.1 | 44.3% | p=0.234 |
|  |  | > Median | 23.8 ± 7.5 | 9.0 - 38.6 | 24.8% |  |
| TNFα | 13.8 | ≤ Median | 30.6 ± 9.3 | 12.5 - 48.7 | 34.8% | p=0.969 |
|  |  | > Median | 23.8 ± 5.5 | 13.0 - 34.6 | 34.4% |  |
| **TGFβ-1** | 86.3 | ≤ Median | n.r. | - | 61.7% | **p=0.012** |
|  |  | > Median | 21.7 ± 4.5 | 12.9 - 30.6 | 12.0% |  |
| TGFβ-2 | 27.8 | ≤ Median | 33.7 ± n.r. | - | 47.9% | p=0.178 |
|  |  | > Median | 23.8 ± 6.1 | 11.8 - 35.9 | 18.8% |  |
| **TGFβ-3** | 7.845 | < Median | n.r. | - | 67.0% | **p=0.006** |
|  |  | > Median | 21.7 ± 1.6 | 18.6- 24.7 | 11.1% |  |

**Median survival shown in months; n.r.: not reached; CI: confidence interval; 3-Y-S: 3-Year-Survival; statistically significant factors are marked in bold; for reason of small sample size standard deviation and confindence interval could not be calculated for all cases**

**B) Prognostic value of cytokines in patients with SCC**

| **Serum factor SCC** | **Median** |  | **Median Survival** | **95% CI** | **3-Y-S (%)** | **p Value** |
| --- | --- | --- | --- | --- | --- | --- |
| IL-1ra | 225.7 | < Median | 16.0 ± 7.4 | 1.5 - 30.5 | 40.0% | p=0.219 |
|  |  | > Median | n.r. | - | 64.2% |  |
| IL-6 | 15.6 | < Median | 23.1 ± n.c. | - | 46.7% | p=0.579 |
|  |  | > Median | n.r. | - | 56.5% |  |
| IL-12 | 53.7 | < Median | 18.8 ± 5.8 | 7.4 - 30.2 | 40.0% | p=0.292 |
|  |  | > Median | n.r. | - | 65.5% |  |
| IL-17 | 336.6 | < Median | 11.6 ± 3.7 | 4.3 - 18.8 | 40.0% | p=0.089 |
|  |  | > Median | n.r. | - | 63.2% |  |
| **CCL11** | 118.8 | < Median | 12.3 ± 3.5 | 5.5 - 19.1 | 32.0% | **p=0.022** |
|  |  | > Median | n.r. | - | 71.8% |  |
| FGF basic | 78.9 | < Median | 14.0 ± 4.8 | 4.7 - 23.3 | 40.0% | p=0.127 |
|  |  | > Median | n.r. | - | 63.2% |  |
| GM-CSF | 12.4 | < Median | 16.0 ± n.r. | n.r. | 46.7% | p=0.503 |
|  |  | > Median | n.r. | - | 56.2% |  |
| **IFN-**γ | 186.1 | < Median | 8.7 ± 2.7 | 3.3 - 14.0 | 26.7% | **p=0.001** |
|  |  | > Median | n.r. | - | 77.4% |  |
| CXCL-10 | 843.1 | < Median | 23.0 ± 8.2 | 7.1 - 39.0 | 44.0% | p=0.424 |
|  |  | > Median | n.r. | - | 60.0% |  |
| MCP-1(MCAF) | 90.6 | < Median | 16.0 ± 4.0 | 8.1 - 23.9 | 36.7% | p=0.118 |
|  |  | > Median | n.r. | - | 66.0% |  |
| MIP1a | 7.3 | < Median | 14.0 ± 4.8 | 4.7 - 23.3 | 40.0% | p=0.149 |
|  |  | > Median | n.r. | - | 63.3% |  |
| MIP1b | 111.5 | < Median | 23.0 ± 8.2 | 6.9 - 39.2 | 43.6% | p=0.467 |
|  |  | > Median | n.r. | - | 60.0% |  |
| Rantes | 18507.6 | < Median | n.r. | - | 60.0% | p=0.318 |
|  |  | > Median | 18.8 ± 5.6 | 7.9 - 29.7 | 42.8% |  |
| TNFα | 63.0 | < Median | 14.0 ± 4.8 | 4.7 - 23.3 | 40.0% | p=0.130 |
|  |  | > Median | n.r. | - | 63.2% |  |
| TGFβ-1 | 33125.4 | ≤ Median | 16.0 ± 6.1 | 4.1 - 28.0 | 35.7 | p=0.179 |
|  |  | > Median | n.r. | - | 67.3 |  |
| TGFβ-2 | 807.4 | ≤ Median | 16.0 ± 4.7 | 6.9 - 25.2 | 33.3% | p=0.069 |
|  |  | > Median | n.r. | - | 72.9% |  |
| TGFβ-3 | 314.2 | ≤ Median | 16.0 ± 9.3 | 0.0 - 34.2 | 41.7% | p=0.410 |
|  |  | > Median | n.r. | - | 59.8% |  |
|  |  |  |  |  |  |  |
|  |  |  |  |  |  |  |
| **Tissue factor SCC** | **Median** |  | **Median Survival** | **95% CI** | **3-Y-S (%)** | **p Value** |
| IL-1ra | 5410.56 | ≤ Median | 20.5 ± 4.5 | 11.6 - 29.3 | 38.4% | p=0.719 |
|  |  | > Median | 11.6 ± 3.2 | 5.4 - 17.8 | 40.0% |  |
| IL-6 | 45.77 | ≤ Median | 20.5 ± 8.6 | 3.7 - 37.2 | 38.4% | p=0.923 |
|  |  | > Median | 14.0 ± 3.0 | 8.2 - 19.8 | 40.0% |  |
| IL-12 | 10.94 | ≤ Median | 20.5 ± 4.6 | 11.5 - 29.4 | 37.9% | p=0.617 |
|  |  | > Median | 11.6 ± 3.9 | 3.9 - 19.3 | 40.0% |  |
| IL-17 | 14.68 | ≤ Median | 16.0 ± 4.6 | 7.1 - 25.0 | 35.2% | p=0.860 |
|  |  | > Median | 12.3 ± 1.1 | 10.2 - 14.4 | 44.4% |  |
| CCL11 | 51 | ≤ Median | 20.5 ± 4.6 | 11.5 - 29.4 | 37.9% | p=0.615 |
|  |  | > Median | 10.4 ± 3.4 | 3.8 - 17.0 | 40.0% |  |
| FGF basic | 430.6 | ≤ Median | 12.3 ± 4.4 | 3.6 - 21.0 | 36.4% | p=0.372 |
|  |  | > Median | 20.5 ± 6.6 | 7.5 - 33.4 | 44.4% |  |
| GM-CSF | 70.29 | ≤ Median | 16.0 ± 2.8 | 10.5 - 21.6 | 27.3% | p=0.655 |
|  |  | > Median | 11.6 ± n.r. | - | 50.0% |  |
| IFN-γ | 28.93 | < Median | 20.5 ± 4.6 | 11.5 - 29.4 | 37.9% | p=0.585 |
|  |  | > Median | 10.4 ± 3.9 | 2.6 - 18.1 | 40.0% |  |
| CXCL-10 | 217.31 | < Median | n.r. | - | 51.1% | p=0.386 |
|  |  | > Median | 11.6 ± 1.5 | 8.6 - 14.6 | 30.0% |  |
| MCP-1(MCAF) | 58.49 | < Median | 20.5 ± 8.5 | 3.9 - 37.1 | 39.0% | p=0.949 |
|  |  | > Median | 12.3 ± 5.4 | 5.4 - 19.2 | 40.0% |  |
| MIP1a | 3.66 | < Median | 20.5 ± 4.5 | 11.6 - 29.3 | 38.4% | p=0.727 |
|  |  | > Median | 11.6 ± 1.5 | 8.6 - 14.6 | 40.0% |  |
| MIP1b | 46.63 | < Median | 16.0 ± 4.0 | 9.4 - 22.7 | 40.9% | p=0.907 |
|  |  | > Median | 12.3 ± 8.0 | 0.0 - 27.9 | 40.0% |  |
| Rantes | 1262.66 | < Median | 20.5 ± 4.5 | 11.6 - 29.3 | 38.4% | p=0.750 |
|  |  | > Median | 11.6 ± 1.5 | 8.6 - 14.6 | 40.0% |  |
| TNFα | 22.48 | < Median | 12.3 ± 1.5 | 9.4 - 15.2 | 20.2% | p=0.193 |
|  |  | > Median | n.r. | - | 60.0% |  |
| TGFβ-1 | 72.2 | ≤ Median | 16.0 ± 5.8 | 4.6 - 27.4 | 27.7% | p=0.396 |
|  |  | > Median | 14.0 ± n.r. | - | 50.0% |  |
| **TGFβ-2** | 28.0 | ≤ Median | 11.6 ± 4.1 | 3.5 - 19.6 | 21.2% | **p=0.038** |
|  |  | > Median | n.r. | - | 60.0% |  |
| TGFβ-3 | 8.71 | ≤ Median | 20.5 ± 5.7 | 9.2 - 31.7 | 38.4% | p=0.814 |
|  |  | > Median | 11.6 ± 2.9 | 5.9 - 17.3 | 40.0% |  |

**Median survival shown in months; SCC: squamous cell carcinoma n.r.: not reached; CI: confidence interval; 3-Y-S: 3-Year-Survival; statistically significant factors are marked in bold; for reason of small sample size standard deviation and confindence interval could not be calculated for all cases**
